# Predictive clinical utility of pre-hospital point of care lactate for transfusion of blood product in patients with suspected traumatic haemorrhage: *derivation of a decision-support tool*

**DOI:** 10.1186/s13049-022-01061-x

**Published:** 2022-12-13

**Authors:** J. E. Griggs, R. M. Lyon, M. Sherriff, J. W. Barrett, G. Wareham, E. ter Avest, Air Ambulance Charity Kent Surrey Sussex

**Affiliations:** 1 Air Ambulance Charity Kent Surrey Sussex, Hanger 10 Redhill Aerodrome, Redhill, RH1 5YP UK; 2 University of Surrey, School of Health Sciences, Priestley Rd, Guildford, GU2 7YH UK; 3 Department of Emergency Medicine, University Medical Center Groningen, Groningen, The Netherlands; 4 University of Bristol, Child Dental Health, Bristol Dental School, Faculty of Health Sciences, Lower Maudlin Street, Bristol, BS1 2LY UK; 5 South East Coast Ambulance NHS Foundation Trust, Neptune House, Gatwick, Surrey, RH10 9BG UK

## Abstract

**Introduction:**

Pre-hospital emergency medical teams can transfuse blood products to patients with suspected major traumatic haemorrhage. Common transfusion triggers based on physiological parameters have several disadvantages and are largely unvalidated in guiding pre-hospital transfusion. The addition of pre-hospital lactate (P-LACT) may overcome these challenges. To date, the clinical utility of P-LACT to guide pre-hospital blood transfusion is unclear.

**Methods:**

A retrospective analysis of patients with suspected major traumatic haemorrhage attended by Air Ambulance Charity Kent Surrey Sussex (KSS) between 8 July 2017 and 31 December 2019. The primary endpoint was the accuracy of P-LACT to predict the requirement for any in-hospital (continued) transfusion of blood product.

**Results:**

During the study period, 306 patients with suspected major traumatic haemorrhage were attended by KSS. P-LACT was obtained in 194 patients. In the cohort 103 (34%) patients were declared Code Red. A pre-hospital transfusion was commenced in 124 patients (41%) and in-hospital transfusion was continued in 100 (81%) of these patients, in 24 (19%) patients it was ceased. Predictive probabilities of various lactate cut-off points for requirement of in-hospital transfusion are documented. The highest overall proportion correctly classified patients were found for a P-LACT cut-point of 5.4 mmol/L (76.50% correctly classified). Based on the calculated predictive probabilities, optimal cut-off points were derived for both the exclusion- and inclusion of the need for in-hospital transfusion. A P-LACT < 2.5 mmol/L had a sensitivity of 80.28% and a negative likelihood ratio [LR−] of 0.37 for the prediction of in-hospital transfusion requirement, whereas a P-LACT of 6.0 mmol/L had a specificity of 99.22%, [LR−] = 0.78.

**Conclusion:**

Pre-hospital lactate measurements can be used to predict the need for (continued) in-hospital blood products in addition to current physiological parameters. A simple decision support tool derived in this study can help the clinician interpret pre-hospital lactate results and guide pre-hospital interventions in the major trauma patient.

## Background

Over the past decade pre-hospital critical care teams and helicopter emergency medical services (HEMS) have developed transfusion protocols and operational capability to transfuse blood products to patients with suspected traumatic haemorrhagic shock [1, 2]. Clinical gestalt in combination with clinical variables such as systolic blood pressure (SBP) and shock index (SI) are used to quantify the severity of haemorrhagic shock and guide transfusion with blood products, both in the pre-hospital- and the in-hospital phase of care [3].

Clinical variables, however, have several disadvantages in guiding pre-hospital transfusion. First, evidence-based cut-off values to guide transfusion are lacking. As a result, pre-hospital transfusion trigger thresholds vary [3, 4]. Second, physiological parameters cannot reliably determine the degree of occult and temporal haemorrhage as they are highly influenced by autoregulatory responses [3, 5, 6]. Third, changes in blood pressure are a late sign of a haemorrhagic shock and by using SBP or SI as transfusion triggers the transfusion decision point (TDP) may be delayed beyond the critical window for effective resuscitation [7]. Finally, other causes mimic the clinical signs of haemorrhagic shock and may confound decision-making, such as traumatic vasoactive head injuries [8]. As a result, the sensitivity and specificity of clinical variables to predict in-hospital transfusion are limited [4, 9–11].

Pre-hospital point of care lactate measurement (P-LACT) has the potential to overcome some of these shortcomings. Lactate formation in major trauma patients is the result of tissue hypoperfusion, resulting in anaerobic glycolysis. Haemorrhage and inadequate ventilation following a traumatic injury can lead to hypovolemia, hypoxaemia and end-organ hypoperfusion, resulting in anaerobic glycolysis and lactate formation. Furthermore, lactate is formed as a result of adrenergic responses to pain and stress [12–15]. Unlike SBP and SI, P-LACT changes are not balanced by counter-regulatory mechanisms and can be measured early at the point of injury [12]. Previous studies have shown that P-LACT can be used to predict both the need for resuscitative in-hospital care in trauma patients and the outcome of traumatically injured patients [1, 9, 10]. To date, the clinical utility of P-LACT to guide blood product transfusion remains unclear [3, 16].

In this study, we aim to investigate how P-LACT can be used to predict the need for (continued) in-hospital blood product transfusion in patients attended by HEMS with suspected major traumatic haemorrhage.

## Methods

### Study design

We performed a retrospective analysis of all trauma patients with suspected major haemorrhage attended by Air Ambulance Kent Surrey Sussex (KSS) in whom a P-LACT was measured between 8 July 2017 (when lactate measurements became available to the service) and 31 December 2019. We aimed to investigate the relationship between clinical variables and measured P-LACT values with the need for (continued) in-hospital blood product transfusion.

### Study setting

KSS HEMS cover three counties in the southeast of England, a region of 7200km^2^ with a resident population of 4.5 million, and a transient population of 8 million. Two doctor-paramedic teams respond 24/7 in either a helicopter or rapid response vehicle from one operational base, attending approximately 2000 patients per year. Tertiary trauma care in the region is offered at four major trauma centres (MTCs). At the time of the study KSS carried four units of O Rhesus negative packed red blood cells (PRBC) in a CRĒDO CUBE™ (Series 4, 2l Insulation 15, VIP Golden Hour) and four units of *Lyoplas*, freeze dried plasma (FDP) on all missions.

Blood products are administered as per KSS Standard Operating Procedure (SOP) to patients showing signs of severe haemorrhagic shock. The decision to transfuse blood products is based on clinical gestalt, considering clinical history, mechanism, physiology and response to resuscitative efforts. P-LACT is measured as per SOP as an adjunct to clinical findings to help *exclude* traumatic major haemorrhage (cut off < 2.5 mmol/L). Where there is a suspicion of major haemorrhage *and* sign of haemodynamic compromise ‘Code Red’ is declared. Code Red activation enables a titrated transfusion of up to four units of O Rhesus negative PRBC and 4 units of FDP; administration of 10 mL Calcium Chloride 10% (after the 2nd unit of PRBC) and Tranexamic Acid (1 g) through a fluid warmer (Belmont Buddy Lite™ or Warrior Lite™). In addition, a ‘pre-alert’ to the receiving hospital triggers a predefined in-hospital major haemorrhage protocol to ensure blood products are immediately available [17, 18]. Full traceability of blood transfusions and compliance with Blood Safety and Quality Regulations (2007) and Medicines and Healthcare Regulatory Agency (MHRA, 2016) is ensured.

Previously published work at KSS in the same patient cohort highlights an average time from 999 to P-LACT of 66 min [12]. Internal service evaluation highlights a 999 to hospital time of 1 h 50 min, putting the HEMS team patient side at between 30 and 45 min.

### Study population

Patients were included in the analysis if they had presumed major haemorrhage as a result of traumatic injuries for which a P-LACT was measured on scene and for which the patient was transported (by air or land) to an MTC. Exclusions consisted of patients with traumatic cardiac arrest (TCA), patients pronounced life extinct (PLE) on scene, patients with potential haemorrhagic shock from a medical aetiology, patients transferred to non-MTCs, inter-hospital transfers and patients < 16 years of age.

### Point of care lactate sampling

Lactate was measured from venous blood prior to sodium chloride (NaCl 0.9%) flush, and drawn into a 2 mL syringe during venepuncture or after insertion of an intravenous (IV) cannula using the NOVA StatStrip® Biomedical Xpress™ point of care test (POCT) Lactate Meter system [12]. Samples were taken prior to the initiation of blood product transfusion. Measurement results were noted, time-stamped and retrospectively recorded in the KSS electronic patient record system (HEMSBase 2.0, Medic One Systems Ltd, UK).

### Outcome measures

The primary endpoint of interest was the accuracy of P-LACT (both as a continuous variable and at various cut-off points) to predict the requirement for *any* (continued) transfusion of blood products in-hospital.

### Data acquisition

Patient demographics, mechanism of injury and nature of injuries, presenting physiology, POCT results (including lactate) and treatments provided by HEMS and other EMS (including blood product) were retrieved from the dedicated KSS electronic patient clinical record. In-hospital transfusion data were obtained from electronic health records of the respective MTC’s and shared with KSS in accordance with existing data-sharing agreements between KSS and the NHS Trusts.

### Ethical considerations

This project was registered with the University of Surrey, and met National Institute for Healthcare Research (NIHR, UK) criteria as a service evaluation. All the data used for this study were routinely collected as part of standard pre-hospital and hospital patient data collection. The project was approved by the KSS Research & Development Committee and conducted in accordance with Strengthening the Reporting of Observational Studies in Epidemiology (STROBE) Guidelines [19].

### Statistical analysis

Descriptive statistics are given as mean [95% CI] or median [IQR]. Comparisons across groups were made using Fisher’s exact test and ANOVA Kruskal–Wallis test where appropriate. Sensitivity, specificity, and positive- and negative likelihood ratios as well as the percentage correctly classified were calculated for P-LACT values in the range between 1 and 10 mmol/L. Finally, predicted probabilities [95% CI] of the occurrence of the defined endpoint were calculated for each of these values using logistic regression analysis.

Missing values are reported in the results section of the manuscript according to the STROBE guideline [11]. A *p* value < 0.05 was regarded as statistically significant. Statistical analyses were conducted using Stata 17.0 and SPSS 26.0.

## Results

### Study population

**Fig. 1:**
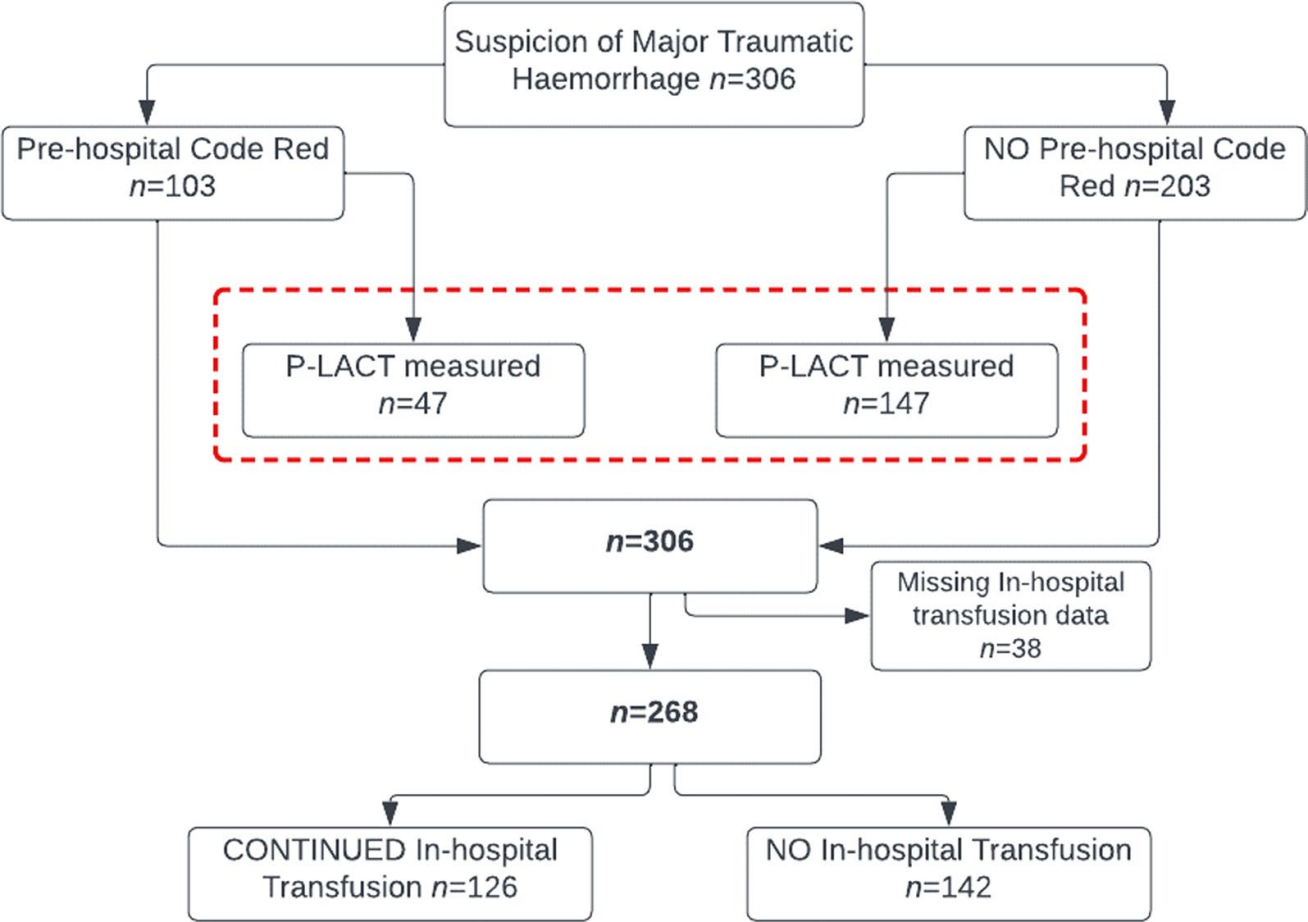
Derived study population and patient inclusion

During the study period KSS attended 306 trauma patients in whom major traumatic haemorrhage was suspected. 103 of these patients had signs of haemodynamic compromise for which a Code Red was declared. Overall, a P-LACT was obtained in a total of 194 patients (47 in whom a Code Red was declared and 147 in whom not). In-hospital transfusion was continued- or started in 126 patients (Fig. 1). 14 of the patients receiving in-hospital transfusion fulfilled criteria for major transfusion, and 31 fulfilled criteria for massive transfusion. P-LACT was not measured in 56 (54%) of the patients with a suspicion of major traumatic haemorrhage.

### Baseline characteristics

Patient demographics, mechanism of injury (MOI), presenting physiology, HEMS interventions and HEMS transfusion of patients attended with suspected major traumatic haemorrhage are presented in Table 1, stratified by the primary endpoint the need for (continued) in-hospital transfusion. Endpoint data were available for 268 (88%) of patients. Patients who needed in-hospital transfusion were more frequently injured in an RTC (72%); had a higher heart rate (106 bpm); a lower systolic blood pressure (88 mmHg); a higher shock index (1.3); a lower GCS (10/15); a higher overall injury severity score (ISS) and average P-LACT of 4.60 mmol/L. HEMS critical interventions were more prevalent in patients who required in-hospital transfusion.

**Table 1:** Population characteristics stratified by need for (continued) transfusion of blood products in hospital

	All patients (n = 306)	In-hospital transfusion (n = 126)	No In-hospital transfusion (n = 142)	*p* value
*Demographics*				
Age, years (SD)	47.4 [21.6]	47.5 [21.7]	47.4 [21.6]	.73
Male, (n [%])	197 [73.5]	90 [71.4]	107 [75.4]	.57
Female, (n [%])	71 [23.2]	36 [28.6]	35 [24.6]	
Missing, (n [%])	38 [3.3]			
*Mechanism descriptors (n, %)*				
RTC	162 [52.9]	91 [72.2]	71 [55.6]	< 0.001
Accidental Injury	20 [6.5]	16 [12.7]	4 [2.8]	
Intentional Self Harm	25 [8.1]	9 [7.14]	16 [11.3]	
Assault	19 [6.2]	8 [6.3]	18 [12.6]	
Fall	30 [9.8]	4 [3.2]	26 [18.4]	
Cyclist	5 [1.6]	0 [0]	5 [3.5]	
Motorcyclist	18 [5.9]	4 [3.2]	14 [9.9]	
Other	8 [2.6]	2 [1.6]	6 [4.2]	
*Presenting physiology*				
TCA (n [%])	15 [8.5]	14 [10.4]	1 [9.2]	< 0.001
HR (mean [SD]) *missing*	91 [25.1] 17	106 [32.6]	98 [29.6]	< 0.001
SBP (mean [SD]) *missing*	106 [34.4] 20	88 [31.8]	120 [29.5]	< 0.001
Shock Index (mean [SD]) *missing*	1.0 [0.5] 27	1.3 [0.6]	0.8 [0.3]	< 0.001
GCS (median [IQR]) *missing*	13 [6–15] 3	10 [3–14] 0	14 [10–15] 3	< 0.001
*P-LACT*				
Presenting P-LACT (mmol/L) (mean [SD]) *missing*	2.9 [2.4] 84	4.60 [2.4]	3.5 [3.5]	< 0.001
*Pre-hospital interventions*				
PHEA (n [%])	101 [36.9]	64 [50.4]	37 [25.5]	< 0.001
Pre-hospital Code Red	103 [39.9]	89 [70.4]	14 [9.2]	< 0.001
Pre-hospital Transfusion	124 [49.0]	100 [79.2]	24 [16.3]	< 0.001
PRBC (units, %) mode [range]		0 [4]	0 [3]	< 0.001
FDP (units, %) mode [range]		1 [4]	0 [4]	< 0.001
PRBC units (n, %)				
*0 units*	169 [60.8]	40 [31.2]	129 [91.5]	.68
*1 units*	44 [17.0]	37 [29.6]	7 [4.3]	
*2 units*	28 [10.1]	24 [19.2]	4 [2.8]	
*3 units*	18 [7.8]	16 [12.8]	2 [1.4]	
*4 units*	9 [4.2]	9 [7.2]	0 [0]	
*Clinical outcome*				
Hospital lactate (mmol/L) *Missing* (n [%])	3.4 [2.6] 204 [66.6]	4.4 [3.0]	2.00 [1.0]	< 0.001
ISS (mean [SD]) *Missing* (n [%])	14 [17–41] 82 [27.8]	33 [23.5–43.0]	22 [13–33]	< 0.001
ICU Length of stay (mean [SD]) *Missing* (n [%])	3 [.00–11.7] 82 [27.8]	4.5 [1.00–16.75]	2 [.0–8.00]	< 0.001
Hospital Length of stay (mean [SD]) *Missing* (n [%])	16 [5.7 – 31.2] 84 [27.45]	18 [3–42]	14 [7–26.0]	.50
Survival to discharge *Missing* (n [%])	144 (47.1) 97[31.7]	57 [44.8]	87 [61.0]	< 0.001

Demographics, proxy injury and physiological parameters: *HR* heart rate, *SBP* systolic blood pressure, *SI* shock index, *GCS* Glasgow Coma Scale, *PHEA* pre-hospital emergency anaesthesia, *PRBC* packed red blood cells, *FDP* freeze-dried plasma, *P-LACT* pre-hospital lactate, *ICU* Intensive Care Unit

### Diagnostic performance of lactate for the prediction of in-hospital transfusion

The negative likelihood ratio [LR−] gives the change in odds of having a diagnosis in patients with a negative test. For example, a −LR of 0.1 would indicate a tenfold decrease in the odds of having a condition in a patient with a negative test result. A -LR of 0.05 would be a 20-fold decrease in the odds of the condition studied.

**Fig. 2:**
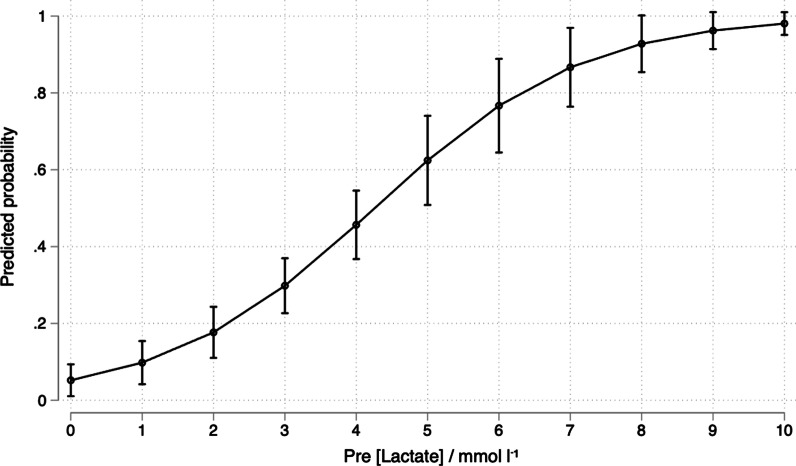
Predictive probabilities (95% CI) of various cut-off points for the need for in-hospital (continued) transfusion. Predicted probabilities and associated 95% CIs of In-hospital transfusion as a function of pre-hospital lactate concentration

Predictive probabilities (95% CI) of cut-off points for every increase in lactate 1 mmol/L for the need for in-hospital transfusion is reported (Fig. 2). The highest overall percentage of correctly classified patients for a single cut-off point was found at the inflection point of the curve, cut-point 5.4 mmol/L. (Table 2). For this cut-point 76.50% of the patients were correctly classified. However, sensitivity of a lactate < 5.4 mmol/L (38.03%) was too low to use as a single cut-off value to exclude the need for in-hospital transfusion.

**Table 2:** Diagnostic performance of P-LACT for prediction of in-hospital blood component therapy in patients with traumatic haemorrhage

Cut-point (mmol/L)	Sensitivity	Specificity	LR+-	LR−	Correctly classified
1	100.00	3.10	1.03	0.00	37.50
2	91.55	31.78	1.34	0.26	53.00
2.5	80.28	51.94	1.67	0.37	62.00
3	69.01	62.78	1.85	0.49	65.00
4	50.34	82.95	3.30	0.52	73.50
5	39.44	93.80	6.36	0.64	74.50
6	22.54	99.22	29.07	0.78	72.00
7	9.86	99.22	12.71	0.90	67.32
8	7.04	100.00	–	0.91	67.50
9	1.41	100.00	–	0.98	65.00
> 9	0.00	100.00	–	1.00	64.50

LR+ is the ratio of the probability of a positive test among the truly positive subjects to the probability of a positive test among the truly negative subjects. The LR– is the ratio of the probability of a negative test among the truly positive subjects to the probability of a negative test among the truly negative subjects

Sensitivity, specificity, and positive- and negative likelihood ratio’s as well as the percentage correctly classified subjects for various P-LACT values in the range between 1 and  > 9.6 mmol/L are represented in Table 2.

The cut-point currently used as an adjunct to *exclude* major haemorrhage (P-LACT < 2.5 mmol/L) had a sensitivity of 80.28%, [LR−] = 0.37 for the prediction of the need for in-hospital transfusion. However, a P-LACT > 2.5 mmol/L only had a specificity of 52.00% and was therefore not useful to identify those patients in whom a code red should be declared and communicated to the receiving hospital. Specificity increased gradually with increasing P-LACT values until a value of 6.0 mmol/L and plateaued thereafter. A P-LACT of 6.0 mmol/L had a specificity of 99.22%, [LR−] = 0.78.

**Fig. 3:**
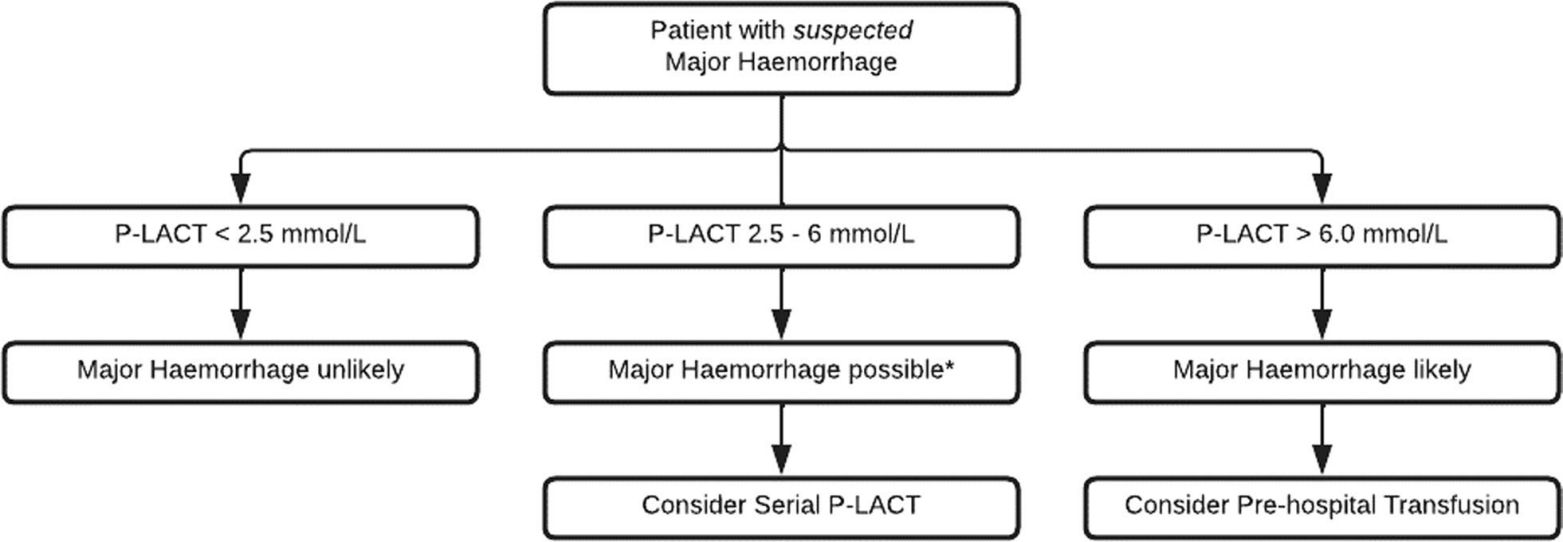
Division of a decision support tool using P-LACT in patients with suspected major haemorrhage. P-LACT, pre-hospital lactate; *Consider alternative causes increased lactate production due to catecholamine release as a result of pain, stress or increased metabolic demand, or due to isolated injuries such as TBI and amputations, or decreased lactate clearance: alcohol intoxication or liver trauma

Based on the above findings a decision support tool was devised for the use of lactate in the pre-hospital phase of care (Fig. 3).

## Discussion

In the present study we demonstrate that pre-hospital lactate measurements can be used to predict the need for (continued) in-hospital blood product. A simple decision support tool can help the clinician to interpret pre-hospital lactate results and to guide transfusion.

When patients are attended by pre-hospital care teams after major trauma, it can be a challenge to determine which patients suffer from major haemorrhage due to their injuries. Often this is obvious from the physical examination (i.e. when external bleeding is present, or when the patient presents in extremis/ peri-arrest). Sometimes, however, this is less clear, as patients may be seen early after their injury, with still relatively preserved haemodynamics. In addition, the clinical picture of major haemorrhage and resultant shock can be mimicked by various conditions, such as vasoactive head injuries [8]. Clinical variables, such as blood pressure and heart rate cannot always discriminate in these instances [4, 10]. Delayed recognition of haemorrhage may delay appropriate treatment beyond the critical window for effective resuscitation [7], and/or omitting a pre-alert to the receiving hospital, leaving clinicians with less time to prepare to receive the patient with the appropriate resources. However, over-transfusion of patients with a suspicion of major haemorrhage may occur based on clinical variables, where a blood transfusion is started in the absence of major haemorrhage, with resultant deleterious effects [6, 20–22]. This is in-line with previous reports on the limited sensitivity and specificity of clinical variables for the prediction of ongoing major haemorrhage [5, 7], even when these variables are incorporated in sophisticated clinical scoring systems, such as the Trauma Associated Severe Haemorrhage Score and Assessment of Blood Consumption Score [3, 23].

In this study, we devise a decision support tool based on P-LACT measurements to aid clinicians at the transfusion decision point. Lactate is a well-known independent prognostic marker of in-hospital mortality in adult trauma patients [24] and previous studies have reported that P-LACT predicts the need for pre-hospital life-saving intervention before- and after admission [15]. The predictive ability of P-LACT for the need of (continued) transfusion requirement in-hospital however has not been studied before.

A P-LACT cut-off value of < 2.5 mmol/L had a sensitivity high enough to exclude the need for in-hospital blood component therapy. This was also a clinically useful cut-off, as 50% of the patients in our cohort had a lactate value < 2.5 mmol/L. P-LACT values > 6.0 mmol/L on the other hand, were highly predictive of the need for (continued) in-hospital transfusion and requirement of blood components. In combination with an appropriate trauma mechanism and/or clinical sign congruent with major haemorrhage, a value > 6.0 mmol/L can almost certainly be used as a trigger to activate a ‘Code Red’ response to initiate pre-hospital transfusion, and to pre-alert the receiving hospital. P-LACT values between 2.5 and 6 mmol/L are not unequivocally diagnostic, which is in-line with previous studies, wherein it was shown that the aetiology of elevated P-LACT levels in trauma patients is multifactorial. Other factors than tissue hypoperfusion, such as increased lactate production due to catecholamine release as a result of pain, stress, or metabolic needs, or a decreased lactate clearance due to alcohol intoxication or liver injuries may play a role. In these instances, serial P-LACT measurements can be considered, especially when transport times to hospital are prolonged and whereby clinicians can aggressively resuscitate a lactate clearance to < 2.5 mmol/L.

Currently, pre-hospital clinicians often transfuse one or more units of blood products to patients in whom major haemorrhage is suspected based on clinical variables. The clinical response is used to establish the need for further transfusion. However, this is not without risk, as transfusion related adverse events (although rare) may occur. Potential deleterious effects may be mitigated, to a certain degree by using serial P-LACT measurements [6]. The proposed algorithm in this study may help pre-hospital clinicians to achieve this. It may also attribute the correct identification of patients who have in-hospital transfusion requirements and may thereby contribute to the improvement of clinical pathways for these patients.

### Limitations

Our study had several limitations. First, selection bias may have influenced our results as P-LACT was not measured in 85/306 patients with suspected major haemorrhage. It is likely that a number of these patients presented in extremis and the completion of P-LACT would prolong the time to transfusion, mandating the crews on scene to prioritize resuscitative interventions above P-LACT measurements. In these patients however it is unlikely that P-LACT will add much in terms of decision-making regarding transfusion requirements. This highlights the challenges of ascertaining the temporality of bleeding in the pre-hospital phase of care. For example, identification and differentiation of the patient whom has active haemorrhage versus the patient who ‘has bled’. Tailoring of transfusion practice to each of these patients is the subject of further research. Further, qualitative research may be able to explore this further in combination with Bayesian theorem. Second, the P-LACT clinical decision tool developed in this study was derived from a relatively limited cohort of 221 trauma patients seen by a single centre. Validation in separate cohorts and other services is warranted to confirm diagnostic accuracy of the proposed P-LACT cut-off points. The decision tool should be tested to see if it results in more correctly classified patients resulting in earlier transfusion in those who need it and withhold transfusion in those who don’t. Third, in-hospital transfusion was used as a surrogate for ongoing haemorrhage. However, it is well known that heuristics and confirmation bias may have influenced the decision to start or continue in-hospital transfusion. Differences in local transfusion policies may have influenced this decision too, as patients were transported to three different MTC’s.

Finally, P-LACT must be adopted within a heuristic approach, where clinical gestalt developed through pattern recognition, clinical observation and perception combine to estimate patient transfusion requirement [25]. We are aware that a single P-LACT is a snapshot of a dynamic process. The clinical utility of P-LACT is likely found in a combination of physiological parameters. Future (Bayesian) prediction modelling to ascertain the value clinicians assign to clinical gestalt at the transfusion decision point may ascertain the adjunctive value of P-LACT in combination with physiological parameters [26] in the temporal management of the bleeding patient.

## Conclusion

Pre-hospital lactate measurements can be used to predict the need for in-hospital blood component therapy. A simple decision support tool derived in this study can help the clinician interpret pre-hospital lactate results and guide the need for blood product transfusion.

## Data Availability

The datasets used and/or analysed during the current study are available from the corresponding author on reasonable request.

## Abbreviations

KSS: Air Ambulance Charity Kent Surrey Sussex
CCP: Critical Care Paramedic
CI: Confidence Interval
EOC: Emergency Operations Centre
FDP: Freeze-dried plasma
GCS: Glasgow Coma Score
HEMS: Helicopter Emergency Medical Service
HR: Heart rate
ICU: Intensive Care Unit
IQR: Interquartile range
MOI: Mechanism of injury
MTC: Major Trauma Centre
NIHR: National Institute for Healthcare Research
PHEA: Pre-hospital emergency anaesthesia
P-LACT: Pre-hospital lactate measurement
PRBC: Packed red blood cells
RTC: Road traffic collision
SBP: Systolic blood pressure
SI: Shock Index
STROBE: Strengthening the reporting of observational studies in epidemiology guidelines
TARN: Trauma audit and research network
TDP: Transfusion decision point
USS: Ultrasound sonography

## References

[1] Holcomb J. Pragmatic, randomized optimal platelet and plasma ratios [Internet]. clinicaltrials.gov; 2019 Feb [cited 2021 Nov 1]. Report No.: NCT01545232. https://clinicaltrials.gov/ct2/show/NCT01545232.

[2] Huang GS, Dunham CM (2017). Mortality outcomes in trauma patients undergoing prehospital red blood cell transfusion: a systematic literature review. Int J Burns Trauma.

[3] Lewis CT, Naumann DN, Crombie N, Midwinter MJ (2016). Prehospital point-of-care lactate following trauma: a systematic review. J Trauma Acute Care Surg.

[4] Petrosoniak A, Hicks C (2018). Resuscitation resequenced: a rational approach to patients with trauma in shock. Emerg Med Clin N Am.

[5] Convertino VA, Howard JT, Hinojosa-Laborde C, Cardin S, Batchelder P, Mulligan J (2015). Individual-specific, beat-to-beat trending of significant human blood loss: the compensatory reserve. Shock Augusta Ga.

[6] Harris T, Davenport R, Mak M, Brohi K (2018). The evolving science of trauma resuscitation. Emerg Med Clin N Am.

[7] Johnson MC, Alarhayem A, Convertino V, Carter R, Chung K, Stewart R (2017). Comparison of compensatory reserve and arterial lactate as markers of shock and resuscitation. J Trauma Acute Care Surg.

[8] Gavrilovski M, El-Zanfaly M, Lyon RM (2018). Isolated traumatic brain injury results in significant pre-hospital derangement of cardiovascular physiology. Injury.

[9] Brown JB, Cohen MJ, Minei JP, Maier RV, West MA, Billiar TR (2015). Pretrauma center red blood cell transfusion is associated with reduced mortality and coagulopathy in severely injured patients with blunt trauma. Ann Surg.

[10] Odom SR, Howell MD, Silva GS, Nielsen VM, Gupta A, Shapiro NI (2013). Lactate clearance as a predictor of mortality in trauma patients. J Trauma Acute Care Surg.

[11] Oyetunji TA, Chang DC, Crompton JG, Greene WR, Efron DT, Haut ER (2011). Redefining hypotension in the elderly: normotension is not reassuring. Arch Surg Chic Ill 1960.

[12] ter Avest E, Griggs J, Wijesuriya J, Russell MQ, Lyon RM (2020). Determinants of prehospital lactate in trauma patients: a retrospective cohort study. BMC Emerg Med.

[13] Bloom BM, Grundlingh J, Bestwick JP, Harris T (2014). The role of venous blood gas in the emergency department: a systematic review and meta-analysis. Eur J Emerg Med Off J Eur Soc Emerg Med.

[14] Baxter J, Cranfield KR, Clark G, Harris T, Bloom B, Gray AJ (2016). Do lactate levels in the emergency department predict outcome in adult trauma patients? A systematic review. J Trauma Acute Care Surg.

[15] Vincent JL, Quintairos e Silva A, Couto L, Taccone FS (2016). The value of blood lactate kinetics in critically ill patients: a systematic review. Crit Care..

[16] Guyette FX, Meier EN, Newgard C, McKnight B, Daya M, Bulger EM (2015). A comparison of prehospital lactate and systolic blood pressure for predicting the need for resuscitative care in trauma transported by ground. J Trauma Acute Care Surg.

[17] Rehn M, Weaver A, Eshelby S, Lockey D (2015). London’s air ambulance: 3 year experience with pre-hospital transfusion. Resuscitation.

[18] Weaver A, Eshelby S, Norton J, Lockey D (2013). The introduction of on-scene blood transfusion in a civilian physician-led pre-hospital trauma service. Scand J Trauma Resusc Emerg Med.

[19] von Elm E, Altman DG, Egger M, Pocock SJ, Gøtzsche PC, Vandenbroucke JP (2007). The Strengthening the Reporting of Observational Studies in Epidemiology (STROBE) statement: guidelines for reporting observational studies. Epidemiol Camb Mass.

[20] Bodnar D, Rashford S, Williams S, Enraght-Moony E, Parker L, Clarke B (2014). The feasibility of civilian prehospital trauma teams carrying and administering packed red blood cells. Emerg Med J.

[21] Miller TE (2013). New evidence in trauma resuscitation—is 1:1:1 the answer?. Perioper Med.

[22] Chaiwat O, Lang JD, Vavilala MS, Wang J, MacKenzie EJ, Jurkovich GJ (2009). Early packed red blood cell transfusion and acute respiratory distress syndrome after trauma. Anesthesiology.

[23] Tonglet ML (2016). Early prediction of ongoing hemorrhage in severe trauma: presentation of the existing scoring systems. Arch Trauma Res.

[24] Salottolo KM, Mains CW, Offner PJ, Bourg PW, Bar-Or D (2013). A retrospective analysis of geriatric trauma patients: venous lactate is a better predictor of mortality than traditional vital signs. Scand J Trauma Resusc Emerg Med.

[25] Cantle PM, Cotton BA (2017). Prediction of massive transfusion in trauma. Crit Care Clin.

[26] Early Identification of Trauma-induced Coagulopathy: Development and Validation of a Multivariable Risk Prediction Model—PubMed [Internet]. [cited 2022 Feb 15]. https://pubmed.ncbi.nlm.nih.gov/31972649/. 10.1097/SLA.0000000000003771 31972649

